# Long non-coding RNA PSMA3-AS1 promotes glioma progression through modulating the miR-411-3p/HOXA10 pathway

**DOI:** 10.1186/s12885-021-08465-5

**Published:** 2021-07-22

**Authors:** Tianzao Huang, Yingxian Chen, Yile Zeng, Chaoyang Xu, Jinzhong Huang, Weipeng Hu, Xiangrong Chen, Huangde Fu

**Affiliations:** 1 Department of Neurosurgery, the Second Affiliated Hospital, Fujian Medical University, Quanzhou, 362000 Fujian China; 2 Department of Neurosurgery, The Jinjiang Municipal Hospital, Quanzhou, Fujian China; 3 Department of Neurosurgery, The Second Nanning People’s Hospital, 13 Dancun Road, Jiangnan District, Nanning, 530031 Guangxi China

**Keywords:** PSMA3-AS1, miR-411-3p, HOXA10, Glioma

## Abstract

**Background:**

Glioma is a common type of brain tumor and is classified as low and high grades according to morphology and molecules. Growing evidence has proved that long non-coding RNAs (lncRNAs) play pivotal roles in numerous tumors or diseases including glioma. Proteasome 20S subunit alpha 3 antisense RNA 1 (PSMA3-AS1), as a member of lncRNAs, has been disclosed to play a tumor-promoting role in cancer progression. However, the role of PSMA3-AS1 in glioma remains unknown. Therefore, we concentrated on researching the regulatory mechanism of PSMA3-AS1 in glioma.

**Methods:**

PSMA3-AS1 expression was detected using RT-qPCR. Functional assays were performed to measure the effects of PSMA3-AS1 on glioma progression. After that, ENCORI (http://starbase.sysu.edu.cn/) database was used to predict potential genes that could bind to PSMA3-AS1, and miR-411-3p was chosen for further studies. The interaction among PSMA3-AS1, miR-411-3p and homeobox A10 (HOXA10) were confirmed through mechanism assays.

**Results:**

PSMA3-AS1 was verified to be up-regulated in glioma cells and promote glioma progression. Furthermore, PSMA3-AS1 could act as a competitive endogenous RNA (ceRNA) for miR-411-3p to regulate HOXA10 and thus affecting glioma progression.

**Conclusion:**

PSMA3-AS1 stimulated glioma progression via the miR-411-3p/HOXA10 pathway, which might offer a novel insight for the therapy and treatment of glioma.

## Background

Glioma is one of the commonest brain tumors originated from glial cells, which can be classified into subtypes such as astrocytoma and glioblastoma according to morphology and histology [1, 2]. Glioma has been divided into four grades by World Health Organization (WHO) classification. Grade-IV glioblastoma is featured by rapid cell proliferation and poor prognosis. The involvements of lncRNAs in the initiation and progression of tumors have been confirmed and proved in recent years, including in glioma [3]. For example, Ni et al. express that GHET1 is overexpressed in glioma and the reduction of it is conducive to hamper the glioma cell activities, thus it is taken as a potential biomarker for glioma [4]. LncRNA AFAP1-AS1 promotes cell invasion and is related to poor prognosis of glioma [5]. At present, surgery, chemotherapy and radiotherapy are the major therapies for glioma, and nanotechnology has gradually been applied for the diagnosis of and treatment for glioma [6]. However, as some tumors have developed resistance against above-mentioned therapies, they will not be affected by medicine intervention. Based on the molecular genetics, a growing number of lncRNAs have been explored and studied, and thus becoming therapeutic biomarkers [7, 8]. Therefore, we aimed at exploring novel biomarkers in the regulatory process of glioma.

Plenty of evidence has showed that lncRNAs act as functional RNA molecules without the ability of encoding protein [9]. The aberrant expression of lncRNAs has been demonstrated to participate in the initiation and progression of tumors and cancers, in which glioma is included [10]. For instance, detailed literature by Qiu et al. has been published recently and disclosed that PSMA3-AS1 is actively involved in the progression of esophageal cancer and serves as a tumor-promoter [11]. However, the role and molecular function of PSMA3-AS1 in glioma remain blurry. What’s more, microRNAs (miRNAs) also have the research value for further study as they can interplay with lncRNAs, and the regulatory relationship between them has been discussed by scientists [12, 13]. Integrated analysis on lncRNA-miRNA-mRNA ceRNA network in cancer development has been made by many studies, including in glioma [14–16] . In this study, we also designed related assays with the aim of exploring the potential ceRNA pattern of PSMA3-AS1 and its effects on glioma progression.

In a word, we focused on the exploration of PSMA3-AS1 expression in glioma cells and how its abnormal expression affects the biological behaviors of glioma cells such as cell proliferation and apoptosis. In addition, we further probed into the regulatory mechanism of PSMA3-AS1 through the ceRNA approach, hoping to provide novel insight into the therapeutic options and treatment for glioma.

## Methods

### Cell culture

Human glial cell line (HEB) was purchased from Qin Cheng Biotechnology Co., Ltd. (Shanghai, China). Human glioma cell lines LN-229 (CRL-2611™) and T98G (CRL-1690™) were all procured from ATCC (Manassas, VA, USA). Human glioma cell line SHG-44 (TCHu 48) was procured from the Cell Resource Center of the Chinese Academy of Sciences (Shanghai, China) and cultivated in RPMI-1640 medium (A4192301, Gibco, Rockville, MD, USA) with 10% fetal bovine serum (FBS; 16,140,071, Thermo Fisher Scientific, Rockford, IL, USA). HEB and LN-229 cells were separately cultivated in DMEM (A4192101, Gibco, Rockville, MD, USA) with 10% FBS (Gibco). T98G was cultivated in Eagle’s Minimum Essential Medium (EMEM) with 10% FBS (Gibco). All of these cells were deposited in 5% CO_2_ at 37 °C. Cell lines have recently been identified by STR cell authentication and tested for mycoplasma contamination.

### Total RNA isolation and quantitative real-time polymerase chain reaction (RT-qPCR)

Total RNA from glioma cells was first extracted with the application of TRIzol Reagent (15,596,018, Invitrogen, Carlsbad CA, USA). Synthesis of complementary DNA (cDNA) was achieved by using PrimeScript™ RT reagent kit (Takara, Shiga, Japan). The acquired cDNA was used for qPCR as per the user guide of SYBR Green PCR Kit (4,309,155, Applied Biosystems, Foster city, CA, USA). Expression of all genes was processed by 2^−ΔΔCt^ method and normalized to GAPDH or U6.

### Cell transfection

Cell samples of LN-229 and T98G were prepared in the 6-well plates for 48 h of transfection with Lipofectamine 3000 (11,668,019, Invitrogen, Carlsbad, CA, USA), in line with the supplier’s protocols. The specific shRNAs targeting PSMA3-AS1 (sh-PSMA3-AS1#1/2) and HOXA10 (sh-HOXA10#1/2) were designed and constructed by GenePharma (Shanghai, China), using the nonspecific shRNAs as negative control (NC). In addition, full-length of HOXA10 cDNA sequence was cloned into pcDNA3.1 overexpression vector (VT8145, YouBio, Guangzhou, China) for plasmid transfection. The knockdown and overexpression of miR-411-3p were separately achieved by transfecting cells with miR-411-3p inhibitor and miR-411-3p mimics, as well as NC inhibitor and NC mimics (all; Ribobio, Guangzhou, China). Experimental procedures were repeated independently for three times.

### EdU assay

1 × 10^4^ transfected cells in 96-well plates were prepared for EdU assay by using BeyoClick™ EdU Cell Proliferation Kit (C10310, Ribobio,Guangzhou, China). To detect cell proliferation, EdU assay kit was added into samples for 2 h at 37 °C, prior to DAPI staining at room temperature for 5 min. The positively stained cells were analyzed under fluorescence microscope (20 × 10) (DMI8, Leica, Wetzlar, Germany). Experimental procedures were repeated independently for three times.

### Colony formation assay

Six hundred cells were collected after being transfected for 48 h and planted in 6-well plates for colony formation. After 14 days, the culture medium was discarded and the cells were washed with PBS for two times. Colonies were fixed by methanol for 15 min, and 0.5% crystal violet staining solution was then added for 10 min at room temperature. Clones more than 50 cells were counted manually. Experimental procedures were repeated independently for three times.

### JC-1 assay

After the transfection, cell samples in PBS were used to detect the change in mitochondrial membrane potential (Δψm). 1 mL of cell suspension was collected and prepared in 6-well plates for culturing for 30 min with 2.5 μg/ml of JC-1 dye (HY-K0601, MedChemExpress, NJ, USA). Finally, the fluorescence microscope was used for analysis. When the membrane potential are normal, JC-1 enters into mitochondria through the polarity of mitochondrial membrane, and forms a polymer emitting red fluorescence due to the increased concentration. In apoptotic cells, mitochondrial transmembrane potential is depolarized, and JC-1 is released from mitochondria with reduced concentration, which is reversed into a monomers emitting green fluorescence. Therefore, we detected the green and red fluorescence qualitatively (offsets of the cell population) and quantitatively (fluorescence intensity of the cell population) to detect changes in mitochondrial membrane potential. Experimental procedures were repeated independently for three times.

### Flow cytometry

1 × 10^6^ transfected cells were plated into 6-well plates for flow cytometry with Annexin V-FITC/PI double staining kit (559,763, BD Biosciences, San Jose, CA, USA) as per user guide. Cells were double-stained in the darkroom for 15 min and then exposed to flow cytometer (92821250S, Beckman Coulter, Kraemer Boulevard Brea, CA, USA) to analyze cell apoptosis. Experimental procedures were repeated independently for three times.

### TUNEL assay

The prepared cells in 96-well plates were first fixed by 4% paraformaldehyde, and then permeabilized with 0.1% Triton-X100, followed by addition of TUNEL kit (12,156,792,910, Roche, Basel, Switzerland). Cell nucleus was visualized using DAPI solution. The apoptotic cells were analyzed using fluorescence microscope (20 × 10) (DMI8, Leica, Wetzlar, Germany). Experimental procedures were repeated independently for three times.

### Subcellular fractionation

The subcellular fraction assay was undertaken by utilizing PARIS™ Kit (AM1921, Invitrogen, Carlsbad, CA, USA). Cell cytoplasm was isolated by adding the cell fractionation buffer, and cell disruption buffer was used to collect cell nucleus. The contents of GAPDH, PSMA3-AS1 and U6 were separately analyzed in two cell fractions with GAPDH and U6 treated as the internal control of cytoplasm and nucleus, respectively.. Experimental procedures were repeated independently for three times.

### FISH assay

FISH assay was implemented in presence of the specific RNA probe to PSMA3-AS1 (Ribobio), following the established protocol. Cell samples were fixed for 15 min, treated with pepsin, and then hybridized with the probe. After washing, DAPI was used to dye cell nucleus and the images were observed under Olympus fluorescence microscope. Experimental procedures were repeated independently for three times.

### RNA pull down assay

RNA pull down assay was implemented with the help of the Pierce Magnetic RNA-Protein Pull-Down Kit (20,164, Thermo Fisher Scientific, Rockford, IL, USA). Cell protein extracts were incubated with the PSMA3-AS1 biotin probe or control probe. After digestion, magnetic beads were added to capture the RNA-protein mixture, followed by elution and RNA extraction. The mixture was analyzed by RT-qPCR. Experimental procedures were repeated independently for three times.

### RNA immunoprecipitation (RIP) assay

Magna RIP™ RNA-Binding Protein Immunoprecipitation Kit was commercially acquired for RIP assay and used as instructed by provider (638,970, Merck, Darmstadt, Germany). Cell lysates were prepared to incubate in RIP buffer with AGO2 antibody (#2897, cell signaling) or control IgG antibody (#5946, cell signaling). Then, the magnetic beads were added to capture the immunoprecipitates, followed by RT-qPCR analysis. The antibodies used in the study were antibodies against IgG, AGO2, PSMA3-AS1, miR-411-3p and HOXA10. Experimental procedures were repeated independently for three times.

### Luciferase reporter assay

Luciferase reporter assay was conducted using pmirGLO dual-luciferase reporter vectors (E1330, Promega, Madison, WI, USA) in line with instruction. Cells were co-transfected with miR-411-3p mimics or NC-mimics and the pmirGLO vectors containing the fragments of PSMA3-AS1 or HOXA10. Forty-eight hours later, the luciferase intensities were determined by Dual-luciferase reporter assay system (E1910, Promega, Madison, WI, USA). Experimental procedures were repeated independently for three times.

### Statistical analyses

All experimental procedures in this study were repeated independently for three or more than three times, and experimental results were given as the mean ± standard deviation (SD). Statistical analyses were achieved in the form of one-way/two-way ANOVA for evaluation among multiple groups with Tukey and Dunnett as the back analysis methods and Student’s *t*-test for evaluation between two groups, using the GraphPad PRISM 6 (GraphPad, San Diego, CA, USA). The significant data were set as *p* < 0.05.

## Results

### PSMA3-AS1 accelerates cell proliferation and suppresses apoptosis in glioma

**Fig. 1:**
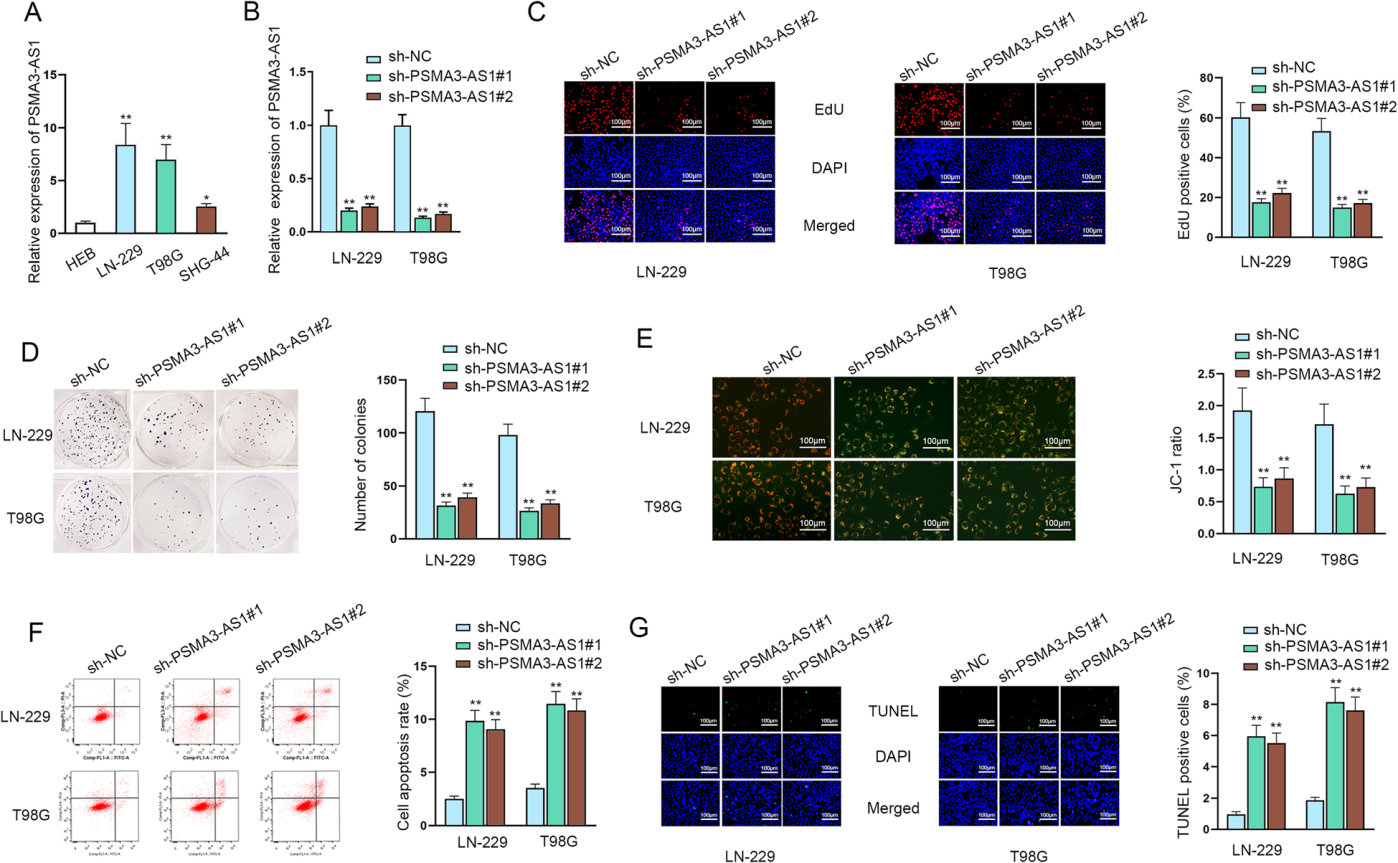
PSMA3-AS1 accelerates cell proliferation and suppresses apoptosis in glioma. **A** The expression level of PSMA3-AS1 was measured by RT-qPCR in glioma cell lines (LN-229, T98G and SHG-44) and normal HEB cell, using one-way ANOVA and Dunnett. **B** RT-qPCR with *t*-test analysis was conducted to assess the interference efficiency of sh-PSMA3-AS1 in glioma cells. **C**-**D** EdU and colony formation assays were adopted to test cell proliferation in glioma upon PSMA3-AS1 silencing. **E**-**G** JC-1, flow cytometry and TUNEL assays were performed to evaluate cell apoptosis after PSMA3-AS1 was inhibited in glioma cells. *P < 0.05, **P < 0.01

To figure out whether PSMA3-AS1 was involved in glioma progression, we applied RT-qPCR to assess the expression of PSMA3-AS1. As demonstrated in Fig. 1a, the expression of PSMA3-AS1 was higher in glioma cell lines (LN-229, T98G and SHG-44) than that in normal human brain astrocytes HEB cell. Since LN-229 and T98G cells have the highest expression of PSMA3-AS1, they were selected for further studies. To further expose the function of PSMA3-AS1 in glioma, sh-PSMA3-AS1#1 and sh-PSMA3-AS1#2 were used to induce PSMA3-AS1 down-regulation (Fig. 1b). After that, EdU and colony formation assays were adopted to evaluate glioma cell proliferation ability, which showed that the proliferative ability of LN-229 and T98G cells was effectively suppressed upon PSMA3-AS1 silencing (Fig. 1c-d). Furthermore, JC-1 assay was conducted to detect cell apoptosis after PSMA3-AS1 was knocked down in glioma cells, and result showed that the JC-1 ratio was declined by PSMA3-AS1 silencing, which meant that the down-regulation of PSMA3-AS1 enhanced cell apoptosis in glioma (Fig. 1e). Experimental data from flow cytometry and TUNEL assays further showed that PSMA3-AS1 silencing promoted glioma cell apoptosis (Fig. 1f-g). Collectively, PSMA3-AS1 is up-regulated in glioma cells and it promotes glioma progression.

### PSMA3-AS1 functions AS a ceRNA for miR-411-3p

**Fig. 2:**
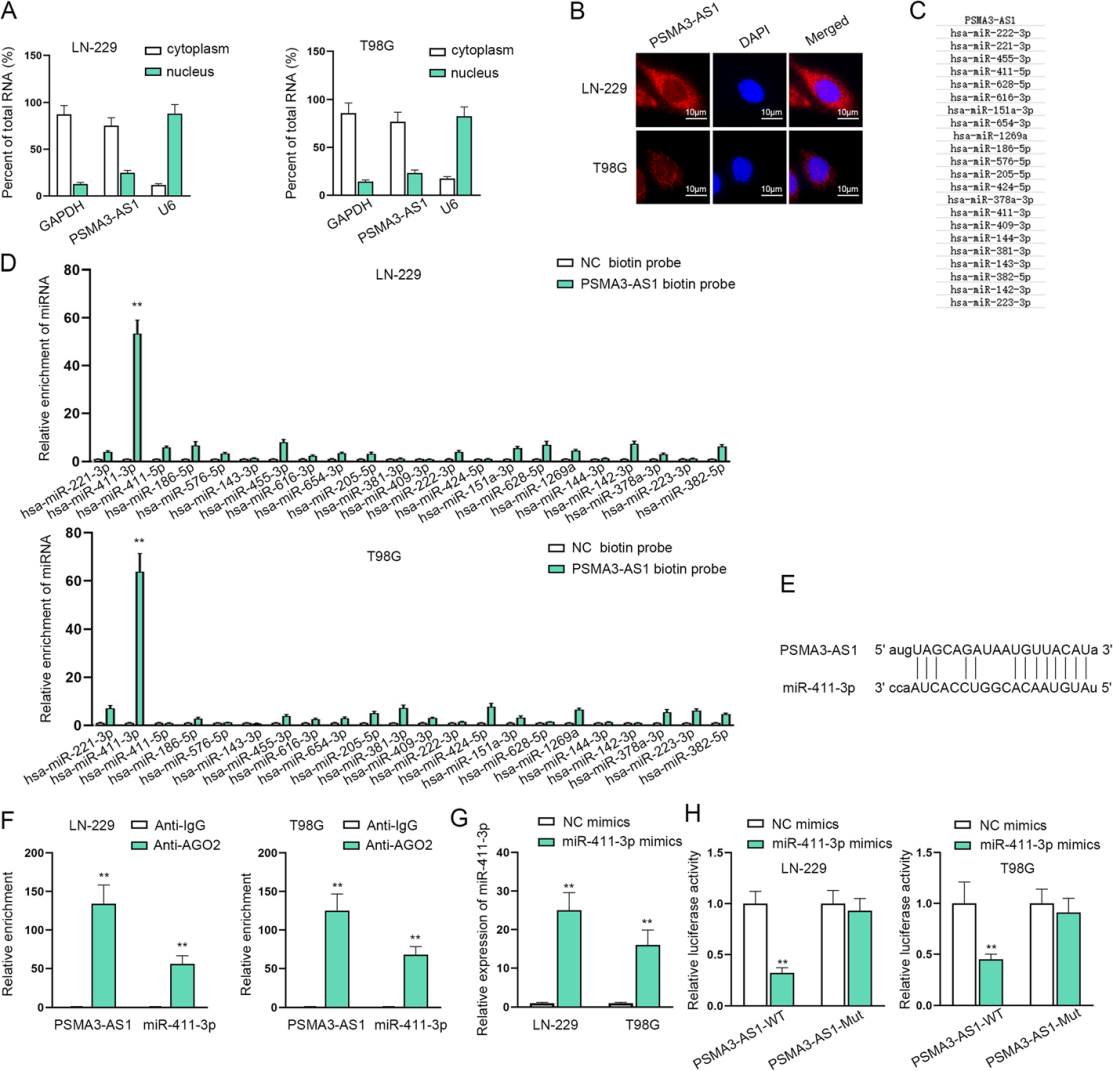
PSMA3-AS1 functions as a ceRNA for miR-411-3p. **A**-**B** Subcellular fractionation and FISH assays were used to confirm the location of PSMA3-AS1 in glioma cells. **C** The possible target genes of PSMA3-AS1 were predicted via ENCORI database. **D** RNA pull down assay was carried out to confirm the interaction between PSMA3-AS1 and potential miRNAs. **E** The binding sites were displayed with the help of ENCORI database. **F** RIP assay was used to test the correlation between PSMA3-AS1 and miR-411-3p. **G** RT-qPCR was implemented to assess the overexpression efficiency of miR-411-3p. **H** Luciferase reporter assay was conducted to test the interaction between PSMA3-AS1 and miR-411-3p using two-way ANOVA and Tukey methods. **P < 0.01

Acting as a ceRNA to sponge and regulate miRNAs is crucial to the mechanism of lncRNAs. Based on this, we first confirmed the location of lncRNA PSMA3-AS1 with the assistance of subcellular fractionation and FISH assays. The results showed that PSMA3-AS1 was intensively distributed in the cytoplasm of LN-229 and T98G cells (Fig. 2a-b). Subsequently, ENCORI (http://starbase.sysu.edu.cn/) database was applied to predict potential miRNAs that could bind to PSMA3-AS1, and 22 candidate genes were selected (Fig. 2c). To further determine the target gene, RNA pull down assay was carried out, and the finding showed that miR-411-3p was obviously enriched with PSMA3-AS1 biotin compared with the control probe group in glioma cells, while no obvious changes were found in other miRNAs (Fig. 2d). Figure 2e also exhibited the underlying binding sites between predicted PSMA3-AS1 and miR-411-3p via ENCORI. Furthermore, it was observed from RIP assay that PSMA3-AS1 and miR-411-3p were both abundant in Anti-AGO2 complex rather than in Anti-IgG group, reflecting the direct interplay between them (Fig. 2f). Besides, RT-qPCR was used to examine the overexpression efficiency of miR-411-3p (Fig. 2g). Finally, it was observed from luciferase reporter assay that PSMA3-AS1 wild type (PSMA3-AS1-WT) expressed lower luciferase activity with miR-411-3p overexpression, while the mutant type of PSMA3-AS1 expressed normally, suggesting the binding ability between PSMA3-AS1 and miR-411-3p (Fig. 2h). Based on the above data, we conclude that PSMA3-AS1 functions as a ceRNA for miR-411-3p in glioma cells.

### MiR-411-3p is able to target HOXA10

**Fig. 3:**
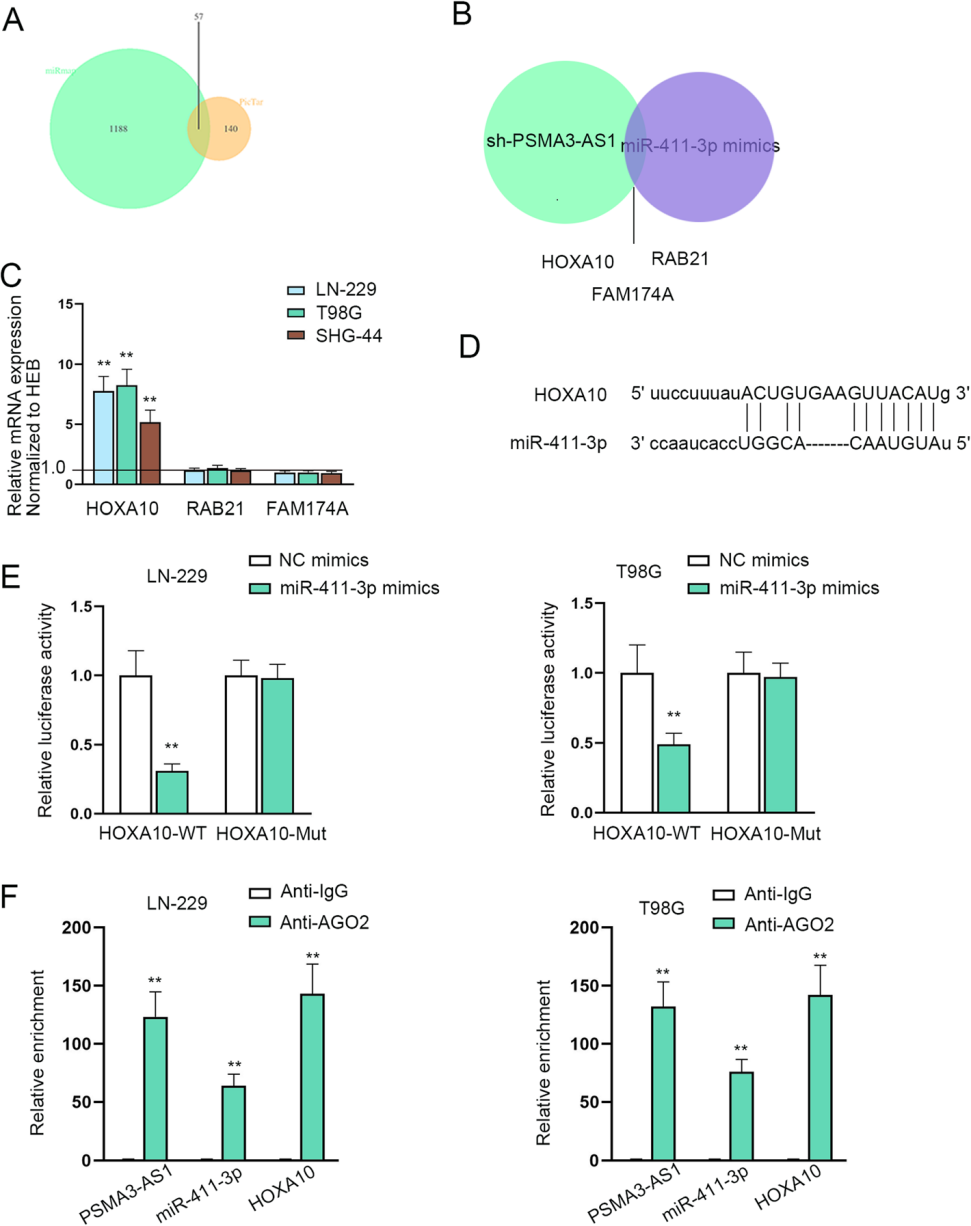
**A** MiR-411-3p is able to target HOXA10. miRmap and PicTar databases were applied to forecast the potential target genes. **B** The expression of possible mRNAs was examined upon PSMA3-AS1 knockdown and miR-411-3p overexpression. **C** The expression of three mRNAs was evaluated via RT-qPCR in glioma cell lines with the help of two-way ANOVA and Tukey analysis. **D** The binding sites of miR-411-3p and HOXA10 were presented by ENCORI database. **E** Luciferase reporter assay was performed to confirm the binding situation between miR-411-3p and HOXA10 using two-way ANOVA and Tukey analysis. **F** The connection among PSMA3-AS1, miR-411-3p and HOXA10 was testified by RIP assay. **P < 0.01

To explore the target gene of miR-411-3p, we applied miRmap (https://mirmap.ezlab.org/) and PicTar (https://pictar.mdc-berlin.de/) databases, and 57 potential targets were selected as shown in Fig. 3a. Afterwards, we performed RT-qPCR assay under the circumstance of co-transfected cells with sh-PSMA3-AS1 and miR-411-3p mimics to further determine the target. The results revealed that HOXA10, RAB21 and FAM174A were with large possibility (Fig. 3b). Based on this, RT-qPCR was again conducted to assess the above three messenger RNAs (mRNAs) expression in glioma cells, which showed that HOXA10 was highly expressed in glioma cell lines compared with the other two mRNAs (Fig. 3c). Therefore, HOXA10 was chosen for further verification. The binding sites between miR-411-3p and HOXA10 were hypothesized through ENCORI database (Fig. 3d). To prove the association and interaction between miR-411-3p and HOXA10, luciferase reporter and RNA pull down assays were utilized. As reflected in Fig. 3e, the luciferase activity of HOXA10-WT was obviously reduced by miR-411-3p mimics compared with NC mimics, while that of HOXA10-Mut was not affected in glioma cells. Moreover, it was confirmed from RIP assay that PSMA3-AS1, miR-411-3p and HOXA10 were co-existed in RNA induce-silencing complex (RISC) (Fig. 3f). Taken together, miR-411-3p is able to target HOXA10.

### HOXA10 silencing hampers glioma cell proliferation and induces cells apoptosis

**Fig. 4:**
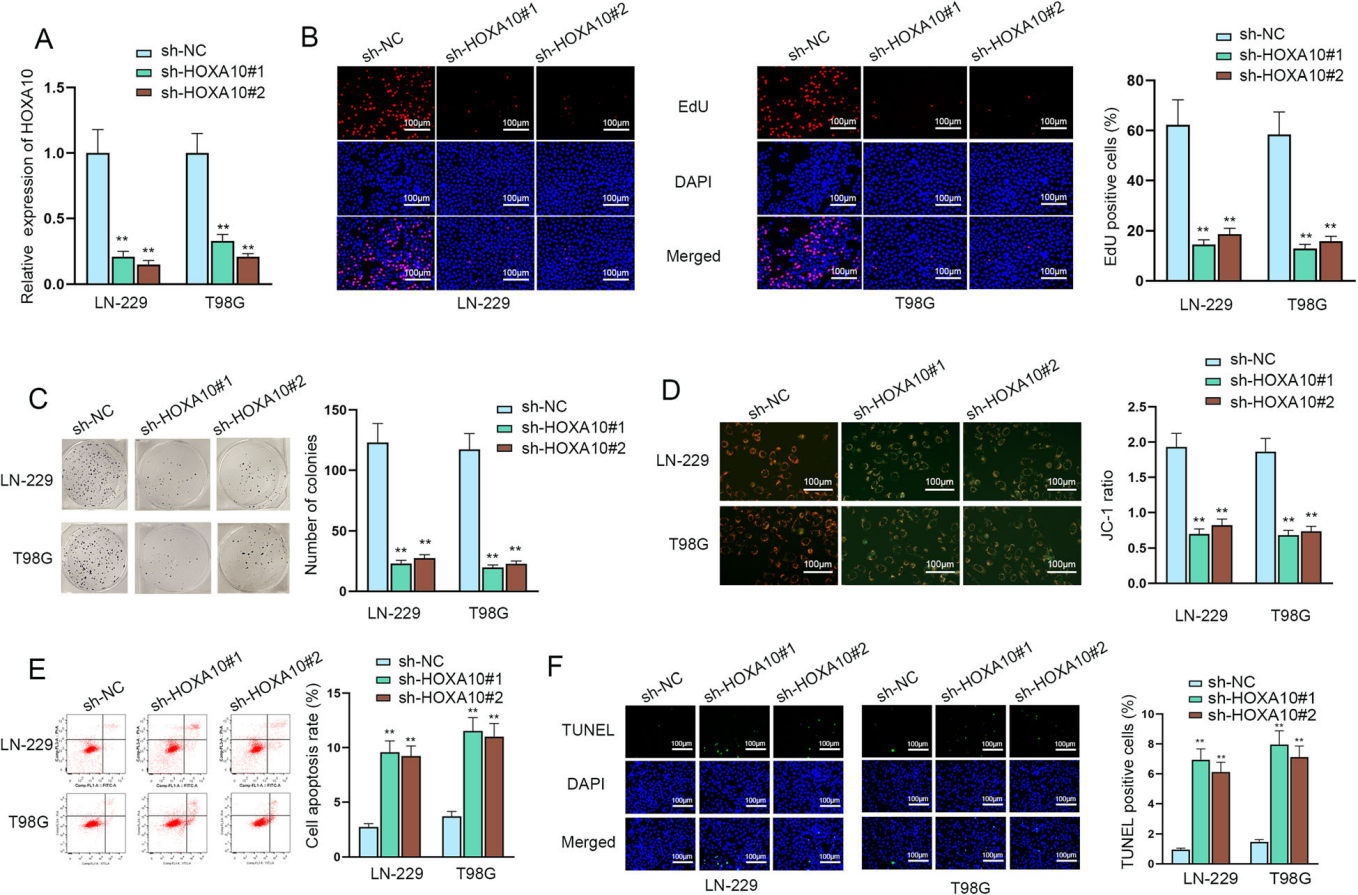
HOXA10 down-regulation hampers glioma cell proliferation and induces cells apoptosis. **A** RT-qPCR was used to assess the inhibitory efficiency of HOXA10 using one-way ANOVA and Tukey. **B**-**C** The proliferative ability of glioma cells upon HOXA10 silencing was measured via EdU and colony formation assays. **D**-**F** The apoptosis capability of glioma cells transfected with sh-HOXA10 was tested by JC-1, flow cytometry and TUNEL assays. **P < 0.01

To further evaluate the role of HOXA10 in glioma progression and how it influences the biological behaviors of glioma cells, LN-229 and T98G cells was transfected with sh-HOXA10#1 and sh-HOXA10#2 and RT-qPCR was used to test the inhibitory efficiency of HOXA10 in glioma cells (Fig. 4a). Subsequently, EdU and colony formation assays were implemented to examine cell proliferation after HOXA10 was knocked down in glioma cells, which turned out that HOXA10 knockdown effectively depressed cell proliferation (Fig. 4b-c). Besides, through JC-1, flow cytometry and TUNEL assays, we discovered that down-regulation of HOXA10 could enhance cell apoptosis in glioma cells (Fig. 4d-f). In a conclusion, HOXA10 promotes the progression of glioma.

### PSMA3-AS1 promotes glioma progression through modulating miR-411-3p/HOXA10 axis

**Fig. 5:**
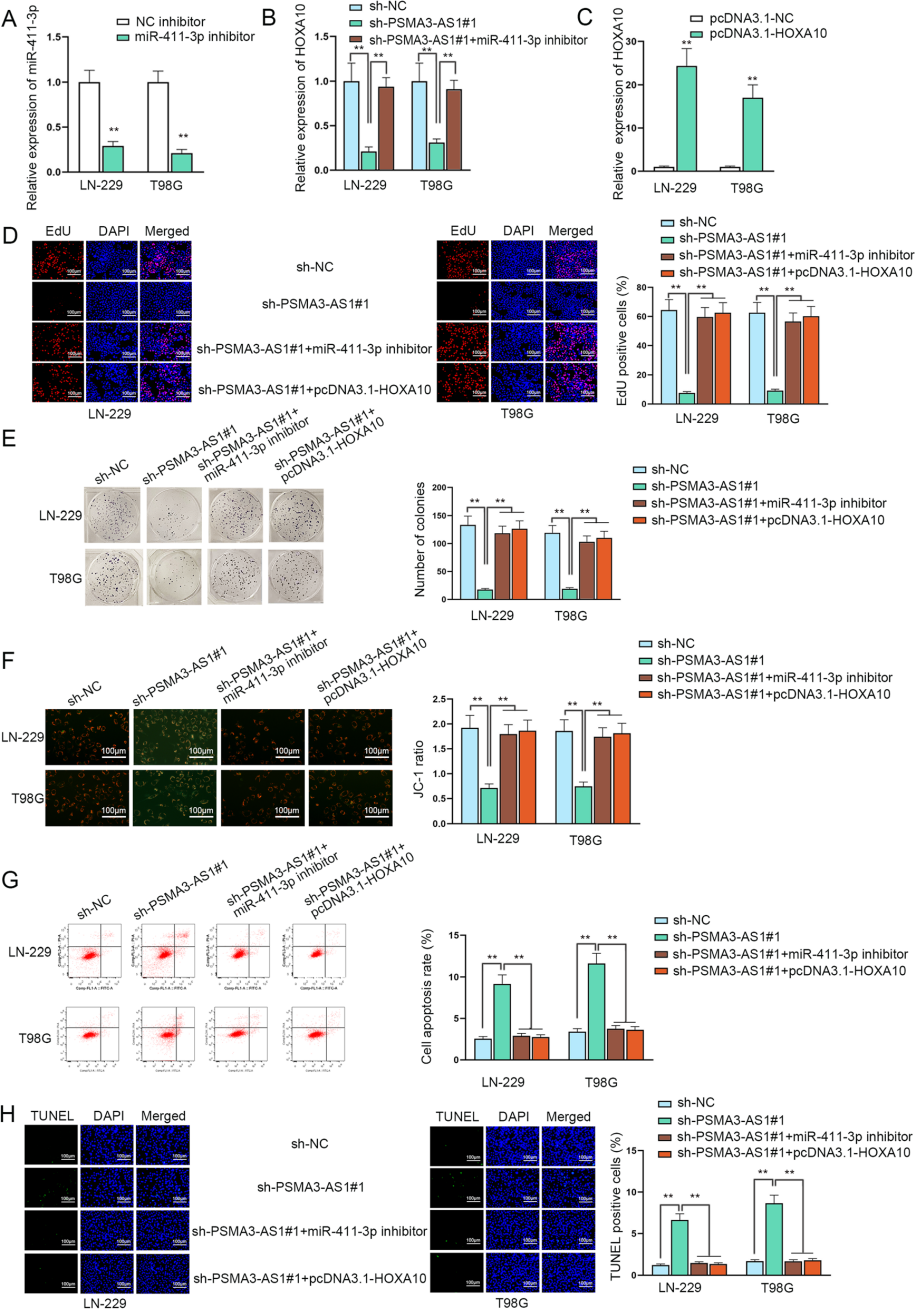
PSMA3-AS1 promotes glioma progression through modulating the miR-411-3p/HOXA10 axis. **A** The interference efficiency of miR-411-3p inhibitor in glioma cells was assessed through RT-qPCR. **B** RT-qPCR was applied to test HOXA10 expression change in response to PSMA3-AS1 silencing and miR-411-3p down-regulation with the help of one-way ANOVA and Tukey analysis. **C** The overexpression efficiency of pcDNA3.1-HOXA10 in glioma cells was tested via RT-qPCR. **D**-**E** Cell proliferation in glioma under different transfection conditions was evaluated by EdU and colony formation assays. **F**-**H** The cell apoptosis ability in different groups was measured via JC-1, flow cytometry and TUNEL assays using two-way ANOVA and Tukey analysis. **P < 0.01. The resolution of all images in the Figure Legends part was at least 300 dpi, and no specific operation was made on enhancing the resolution of individual images

In the last step, a series of rescue assays were designed to confirm the interaction among PSMA3-AS1, miR-411-3p and HOXA10 in glioma. Firstly, the inhibitory efficiency of miR-411-3p in glioma cells was examined via RT-qPCR (Fig. 5a). Then, we divided experimental groups into sh-NC, sh-PSMA3-AS1#1 and sh-PSMA3-AS1#1 + miR-411-3p inhibitor and carried out the following assays. It was shown from Fig. 5b that HOXA10 was primarily cut down by PSMA3-AS1 down-regulation but later rescued by miR-411-3p silencing, which suggested that PSMA3-AS1 could positively regulate HOXA10 via miR-411-3p, and thus the PSMA3-AS1/miR-411-3p/HOXA10 regulatory axis was successfully constructed. After that, how this axis influenced the behavior of glioma cells such as proliferation and apoptosis was studied through related rescue assays. First of all, the overexpression efficiency of HOXA10 was detected by RT-qPCR (Fig. 5c). Next, the rescue assays were respectively carried out, including EdU, colony formation, JC-1, flow cytometry and TUNEL assays. It was disclosed in Fig. 5d and e that the proliferative ability of glioma cells was initially restrained due to the silencing of PSMA3-AS1 while recovered by miR-411-3p silencing or the overexpression of HOXA10. Inversely, silencing PSMA3-AS1 accelerated glioma cell apoptosis, while this effect could be countervailed by miR-411-3p knockdown or HOXA10 up-regulation (Fig. 5f-h). To conclude, PSMA3-AS1 promotes glioma progression through modulating the miR-411-3p/HOXA10 axis.

## Discussion

As one of the commonest type of brain tumors, glioma has been divided into four grades by WHO, and Grade-IV glioblastoma is characterized by rapid cell proliferation as well as poor prognosis. LncRNAs have been illustrated to be involved in the regulation of cancers and glioma is included. PSMA3-AS1 has been disclosed to be actively involved in the progression of esophageal cancer in which it plays tumor-promoting function, while its detailed function and mechanism in glioma remains to be explored. In this study, PSMA3-AS1 was testified to be up-regulated in glioma cells, and then loss-of-function assays were carried out to verify the effects of PSMA3-AS1 on glioma cell proliferation and apoptosis. Results showed that PSMA3-AS1 down-regulation had anti-proliferative and pro-apoptotic effects on glioma cells. On the whole, PSMA3-AS1 was regarded as a carcinogenic gene in glioma.

LncRNAs have been illustrated to exert oncogenic roles as a ceRNA in glioma [17, 18]. In this study, miR-411-3p was selected and verified to be the target gene of PSMA3-AS1 and HOXA10 was identified as the downstream target of miR-411-3p, thus forming the PSMA3-AS1/miR-411-3p/HOXA10 axis. As for miR-411-3p, Halvorsen AR1 et al. show that miR-411-3p is closely associated with the overall survival rate of patient with lung cancer [19] as well as ovarian cancer [20]. HOXA10, as a member of homeobox (HOX) genes, promotes tumor progression in multiple cancers. For example, in colorectal cancer, overexpression of HOXA10 results in low survival rate and forecasts poor prognosis [21]. Similarly, HOXA10 promotes proliferation, migration and invasion in oral squamous cell carcinoma [22]. Besides, gastric cancer [23] and breast cancer [24] and some other cancers have close connection with HOXA10. Back to this current research, HOXA10 silencing was proven to depress glioma cell proliferation while inducing cell apoptosis, indicating that HOXA10 played as an oncogene in glioma. It is worth noting here that HOXA10 has been reported to be an oncogene in glioma before [25] and this finding is consistent with the conclusion of our study, which suggests the research value of HOXA10 in the regulation of glioma progression in the future.

## Conclusions

In conclusion, this study initially uncovered that PSMA3-AS1 could serve as an oncogene in glioma progression by sponging miR-411-3p to regulate HOXA10. It is also the first time that we discovered PSMA3-AS1 may potentially act as a novel biomarker and therapeutic target for glioma treatment.

## Data Availability

Not applicable.

## Abbreviations

lncRNAs: Long non-coding RNAs
mRNAs: Messenger RNAs
miRNAs: MicroRNAs
ceRNA: Competing endogenous RNA
PSMA3-AS1: Proteasome 20S subunit alpha 3 antisense RNA 1
HOXA10: Homeobox A10
ATCC: American Type Culture Collection
DMEM: Dulbecco’s modification of Eagle’s medium
RPMI: Roswell Park Memorial Institute
FBS: Fatal Bovine Serum
RT-qPCR: Reverse transcription quantitative real-time polymerase chain reaction
GAPDH: Glyceraldehyde-3-phosphate dehydrogenase
EdU: 5-Ethynyl-2′-deoxyuridine
DAPI: 4′,6-diamidino-2-phenylindole
TUNEL: Terminal deoxynucleotidyl transferase (TdT) dUTP Nick-End Labeling
FISH: Fluorescence in situ hybridization
RIP: RNA immunoprecipitation
WT: Wild-type
Mut: Mutant
SD: Standard deviation
ANOVA: Analysis of Variance
